# Mixtures of Lubricants and Ecological Refrigerants under Starved Lubrication Conditions

**DOI:** 10.3390/ma15217747

**Published:** 2022-11-03

**Authors:** Kasper Górny, Arkadiusz Stachowiak, Przemysław Tyczewski, Wiesław Zwierzycki

**Affiliations:** Institute of Machines and Motor Vehicles, Poznan University of Technology, 60-965 Poznan, Poland

**Keywords:** lubricating properties, starved lubrication, oil–refrigerant mixture, R452A refrigerant

## Abstract

The presented results show that the presence of refrigerant significantly deteriorates the lubricating properties of compressor oil under starved lubrication conditions (with a small amount of oil). The change can be 40–120% compared to the properties of the oil alone. Additionally, in the group of oils that are substitutes (operational alternatives) compatible with a given refrigerant, the effect of the refrigerant on the lubricating properties varies. The differences can be as much as 25%. In order to evaluate and properly select compressor oils for the refrigerant, the lubricating properties should be tested in a mixture with the refrigerant under conditions similar to actual operation. Such an evaluation of lubricating properties is made possible by the author’s method of testing the wear of the block-on-ring friction node. The obtained rankings of lubricating properties for oils (due to the wear volume) can provide good guidelines for the suitable selection of a lubricant for refrigeration compressors (especially for new, environmentally friendly refrigerants, such as R452A). The research was carried out for mixtures of zeotropic refrigerants (R404A, R452A) with polyester oils (POE) and natural refrigerant (R600a) with mineral oils (MO). In each group of refrigerants, different mechanisms of oil–refrigerant mixture formation occur. Each refrigerant was tested with three different compressor oils recommended for each other for alternative uses in refrigeration systems.

## 1. Introduction

In recent years, there have been significant changes in using refrigerants for various practical applications. European legal regulations have been mainly based on ecological measures such as ODP and GWP. First, the refrigerants that deplete the ozone layer (with ODPs higher than 0) have been eliminated from use [1]. Subsequently, where possible, the use of refrigerants that have a potent greenhouse effect and high global warming potential has been reduced [2]. The abovementioned changes lead to the necessity of seeking lubricants suitable for new ecological refrigerants. 

Previous articles [3,4] prove that damage to refrigeration compressors is largely caused mechanically, mainly because of inefficient lubrication, i.e., lack of oil, its solution with refrigerant and insufficient quantity of oil. 

Starved lubrication occurs in cases where a low amount of lubricant is present in a friction node, which should enable the formation of a permanent boundary layer. Starved lubrication is often a way of lubricating machine friction pairs. When starved lubrication is intentionally forced, the following lubrication types may be identified: drip lubrication, oil–mist lubrication and oil–air lubrication [5]. Generally, it has been noticed that starved lubrication results in decreased thickness and range of oil film and increased temperature in the friction pair area [6,7,8]. It is also worth noting that starved lubrication compared to full lubrication may lead to much more serious damage [9,10]. Starved lubrication is not an assumed lubrication method at the design stage for refrigeration compressors, but it is a consequence of the specific work of refrigeration systems and is both an undesirable and unavoidable operational situation.

Previous tribological tests aimed at evaluating refrigeration compressor lubricants made it partially possible to represent the operational problems of real equipment. In broad terms, the papers [11,12,13,14,15,16,17,18,19,20,21,22,23,24,25,26,27] attempted to evaluate various lubricants under the circumstances where oil was diluted with refrigerant, and the quantity of lubricant was sufficient. Some papers present research ideas and the results of wear tests to allow evaluation of the lubricating properties evaluation oil/refrigerant mixtures [28,29,30,31,32].

Considerably less research has been carried out on extreme operational situations, such as lack of oil or its insufficient amount in friction pairs [11,18,20,24,33,34]. In general, lack of oil in the friction pairs of a refrigeration compressor may cause metal-to-metal contact. Due to an enormous increase in the force of friction, such a situation may result in large energy losses and destructive damage. Lack of oil in a friction pair of a refrigeration compressor is the most undesirable operational situation. 

Lack of oil or its insufficient amount in friction pairs may be caused by too short work cycles of a refrigeration compressor, excessive oil foaming and long periods of the minimum filling of the compressor with oil when the diameters of refrigeration system tubes are incorrect [11,20,24]. An insufficient amount of oil in friction pairs may result in starved lubrication conditions. Starved lubrication may also occur in refrigeration compressors during the turning on or turning off the equipment [18,19]. 

Test results for lubricating oil and refrigerant mixtures under starved lubrication conditions have been presented in several articles [20,24,33]. The authors of [24] used a model pin-on-disc type stand. Most of the tests were carried out with polyalkylene glycol (PAG) oil and R134a refrigerant. Polyester oils (POE) that are compatible with the refrigerant were also used in several tests. Starved lubrication conditions were obtained by applying various quantities of lubricant onto the surface of the upper part of the sample (disc) with the use of a lubricant supply system mounted in the test chamber. Lubricant, after preliminary mixing with liquid refrigerant, was sprayed in the form of oil mist into the friction area through a special nozzle. Spraying was carried out continuously and the tests were performed under steady conditions. All the tests were conducted at a refrigerant pressure equal 0.17 MPa and a temperature of 12 ± 1 °C, i.e., the operating conditions of friction node in swashplate refrigeration compressors using R134a refrigerant. The coefficient of friction values and roughness profiles were checked.

In the work [20], the mixture of mineral oil (MO) and R600a refrigerant was tested. One of the goals was to compare weight loss after tests in air and R600a. In addition, an oil–refrigerant mixture in starved lubrication conditions was tested. A fixed quantity of lubricant (100 mg) was applied under pin sample of pin-on-disc wear tester. R600a refrigerant was then continuously supplied to the test chamber. The refrigerant pressure and the formation time of oil/refrigerant mixture were not provided by the authors. The tests were performed at an ambient temperature of 23 ± 1 °C. The coefficient of friction values and the wear of a disc-shaped sample were analyzed. Tests where air was used instead of refrigerant were also carried out for comparison. Based on the figures presented in [20], it can be concluded that the friction coefficient in tests with air is 50–90% higher than in tests with R600a. The results of the tests showed about twice as much weight loss when using R600a refrigerant versus tests in air.

In order to obtain starved lubrication conditions, the authors of [11,33,35] applied small amount of lubricant (weighing approx. 22 mg) to one of friction node samples. That quantity should be enough to provide a boundary layer. Similar conditions for ensuring starved lubrication were proposed in [36]. In this experiment, three drops (75 mg) of lubricant were used and the amount was quantified based on the weight of the lubricant on the surface. In [37], lubricant was uniformly sprayed onto the surface of the element in an amount of 0.4 μL/cm^2^ using precision spraying equipment. Another approach also involved the continuous application of lubricant, in this case in an amount of 25–35 mg/min [38].

Until recently, R404A was a quite commonly used HFC refrigerant for commercial refrigeration equipment in small and large systems. The refrigerant was widely used, inter alia, in the food industry and in transport refrigeration. R404A refrigerant has considerable greenhouse effect potential (GWP = 3922) which is why replacements with a low GWP and similar efficiency parameters are currently being sought. The replacements for R404A include: R-407A [39], R-407F [40], R-407H [41], R-448A [42,43], R-449A [44] and R-452A [26,45] with GWPs ranging from 1387 to 2140. R452A is one of the most promising substitutes for R404A refrigerant—especially in the food industry and in transport refrigeration. Currently the refrigerant is used by a number of companies due to its relatively low discharge temperatures which reduces the risk of operational problems in refrigeration compressors. The composition of R452A refrigerant has been developed to best match the pressure-enthalpy and temperature-entropy characteristics [46]. The components of R452A refrigerant are: 11%—R32, 59%—R125 and 30%—R1234yf. The composition of R452A is based on hydrofluoroolefins and has a lower global warming potential than R404A (GWP = 1945). In [47,48], the thermodynamic and operational properties of R404A and R452A refrigerants were compared. The miscibility of R452A refrigerant with various lubricants under the operational conditions of refrigeration systems was also discussed. So far, R452A refrigerant has not been well investigated in terms of its tribological properties. The study on the lubricating properties of the mixtures of polyether vinyl (PVE) oils with, among others, R404A and R452A refrigerants was presented in [26]. While in [49], an attempt was made to assess, among others, the tribological properties of polyester oils (POE) and R452A refrigerant mixtures in full lubrication conditions. 

The main objectives of the article are:

1. Identifying the influence of the refrigerant on the lubricating properties of oil–refrigerant mixtures in starved lubrication conditions (very small amount of oil in a friction pair),
2. A comparative assessment of the lubricating properties of oil–refrigerant mixtures in starved lubrication conditions for groups of oils that are substitutes (operational alternatives) compatible with a given refrigerant.

In pursuit of the former objective, a method of testing the lubricating properties of an oil–refrigerant mixture in starved lubrication conditions was proposed. In this regard, an original test stand was used, and a test procedure performed in full lubrication conditions was adapted (for detailed descriptions see [28]). The article presents the results verifying the effectiveness of the proposed concept.

In order to achieve the latter objective, three polyester (POE) oils with R404A refrigerant and R452A refrigerant and three mineral oils (MO) mixed with R600a refrigerant were tested. The diverse refrigerants were used to map the different mechanisms of an oil–refrigerant mixture formation. In the case of R600a natural refrigerant, it is a slow process determined by diffusion. R600a refrigerant is considered to be an ecological substitute for R134a refrigerant in small refrigeration equipment [50]. The formation of mixtures for R404A and R452A synthetic refrigerants is faster and relates to gravity effects. The lubricating properties of the group of oils (POE) were tested in a mixture with R404A refrigerant that had been used for years and its current substitute R452A refrigerant (which is more ecological). The test results were used to answer whether the experience in using R404A refrigerant gained over the years may be used for selecting oils for the new R452A refrigerant. 

## 2. Materials and Methods

Three mineral oils (MO1, MO2 and MO3) with VG 32 viscosity grade suitable to work with isobutane R600a and three polyester oils (POE1, POE2 and POE3) with VG 32 viscosity grade dedicated to work with R404A and R452A refrigerants were selected to compare lubricating properties in starved lubrication conditions. The selected oils are substitutes for each other for alternative uses in refrigeration systems. They are commercial products from different manufacturers with the same performance characteristics (including viscosity). Properties of investigated polyester oils and refrigerants are shown in Table 1 and Table 2.

**Figure 1:**
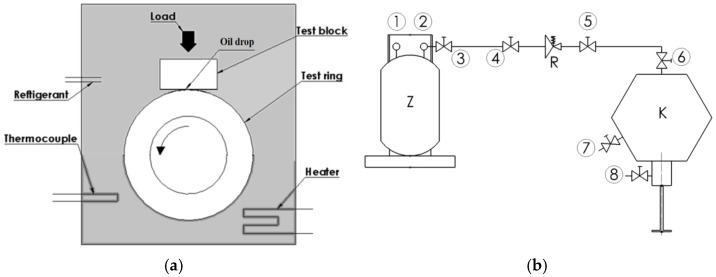
(**a**) Scheme of the block-on-ring type wear tester; (**b**) set-up for supplying refrigerant: Z—refrigerant tank; R—pressure regulator; K—high pressure chamber; 1–8—ball valves.

The wear of a sample in the shape of a model block-on-ring pair (Figure 1a) was used for the quantitative assessment of the lubricating properties of refrigeration compressor oil in a mixture with refrigerant in starved lubrication conditions. Less wear of the block will correspond to better lubricating properties of the mixture. That sample type was selected due to its similarity to the geometry of the crankshaft elements of reciprocating refrigeration compressors. On the test stand (Figure 1b), one may reproduce parameters characteristic for starting the compressor after an extensive stand-still period. This is when the concentration of the refrigerant in the mixture inside the compressor is the highest.

According to the concept proposed by the authors, the following test parameters must be determined to assess the lubricating properties of an oil–refrigerant mixture:

- Refrigerant pressure in the test chamber (p_s_),
- Tribological test duration time (τ_t_),
- Oil–refrigerant mixture formation time (τ_m_).

A schematic method of determining the parameters is presented in Table 3, while a detailed description of determining the parameters can be found in [30,49]. Test parameters should be determined individually depending on the refrigerant and oil to be tested. The order in which the parameters are determined is important, and it is expressed by the yellow markings in Table 3. First, the refrigerant pressure in the test chamber (p_s_) should be determined. Next, after tests with just oil, the tribological test duration time (τ_t_) is specified. In last step the oil–refrigerant mixture, formation time (τ_m_) is determined after tests with the oil–refrigerant mixture.

The formation of an oil–refrigerant mixture in a special chamber (Figure 1b) is the first stage of the proposed test method (Table 3). The adapted test concept involves copying the conditions prevailing in a compressor after an extensive stand-still period. The pressure (p_s_) was determined on the basis that refrigerant saturation pressure at an ambient temperature (approx. 23 °C) is thus maintained in the test chamber. The wear test duration (τ_t_) should be long enough to obtain a clear sample wear. The main tests of the lubricating properties of an oil–refrigerant mixture are preceded with preliminary test series at various values of the parameter (τ_t_) until the assumed minimum wear value of 0.5 mm^3^ [30] is obtained. The preliminary tests are carried out with the use of oil which should be impacted upon by air in order to provide a predetermined pressure (*p_s_*) in the test chamber. The last parameter of the test method is oil–refrigerant mixture formation time (τ_m_). It should be long enough to stabilize the mixture composition (maximum refrigerant content in the oil). The parameter (τ_m_) is determined on the basis of the results of preliminary wear test series (for a predetermined test duration time (τ_t_)) that follow different mixture formation times. Stabilisation of the mixture composition occurs when the extension of mixture formation time does not significantly change the value of sample wear [30,49].

A pressure (p_s_) of approx. 0.21 MPa was maintained in the test chamber while performing tests necessary for evaluating the lubricating properties of mineral (MO) oils and their mixtures with isobutane (R600a). Pressures (p_s_) of 1.10 MPa for R404A refrigerant tests and 1.20 MPa for R452A, respectively, were maintained in the test chamber while testing the lubricating properties of polyester (POE) oils and their mixtures with the refrigerants. The values correspond to the saturation pressure of the tested refrigerants at a temperature of 23 °C. 

The same value of oil–refrigerant mixture formation time (τ_m_) for the tests in starved lubrication conditions (one drop of oil) as in the earlier tests in full lubrication conditions (oil filled half of the chamber—Figure 1a) was adopted. The method of determining the parameter and the results were described in [30,49]. The time sufficient for the formation of a saturated oil–refrigerant mixture in a large volume should also ensure saturation of the oil drop with refrigerant in starved lubrication conditions. The wear test duration time (τ_t_) for the tests under starved lubrication conditions was determined in a series of independent tests. The results are presented in Section 3.1.

The test parameters for lubricating properties of mineral oils and R600a refrigerant mixtures and polyester oils in mixtures with R404A and R452A refrigerants in starved lubrication conditions are presented in Table 4. 

During the main tests evaluating lubricating properties, samples were ultrasonically cleaned in acetone for 15 min before each test. Then, the samples were mounted in the test chamber. In the next step, air was removed from the test chamber and then a drop of oil was placed on a ring-shaped sample (about 30 mg). Then refrigerant of a selected pressure (p_s_) was supplied to the chamber and the conditions were maintained for a specified time (τ_m_). When the mixture was formed, a wear test of a duration time (τ_t_) was carried out. After each wear test, the width trace of wear was measured on the block-shaped sample and the wear volume was calculated [30,49].

The moment of force was measured during the tests. Next, the coefficient of friction was calculated on the basis of the following formula based on brake system [51]:

(1) $$\mu =\frac{M}{P\;r}$$

where *μ* is coefficient of friction (-), *M* is moment of force (Nm), *P* is load (pressure force) (N) and *r* is radius of the ring (m).

Table 5 provides a summary of the performed test series. Three wear tests were performed for each of the test series. 

During the 1 and 9 (air) and 5, 13 and 17 (only refrigerants, R600a, R542A and R404A respectively) series tests, the pressure (p_s_) was maintained in the chamber, but no lubricant was supplied. For the 2–4 (MO), 6–8 (MO/R600a), 10–12 (POE), 14–16 (POE/R452A) and 18–20 (POE1/R404A) series tests, the pressure (p_s_) was maintained in the chamber, the air was supplied in the 2–4 and 10–12 series and the appropriate refrigerant was supplied in the 6–8, 14–16 and 18–20 series, respectively. A small quantity of the appropriate lubricant (one drop) was introduced to the friction pair following the guidelines listed in Table 4 and Table 5 in all series except series 1, 5, 9, 13 and 17.

## 3. Results

**Figure 2:**
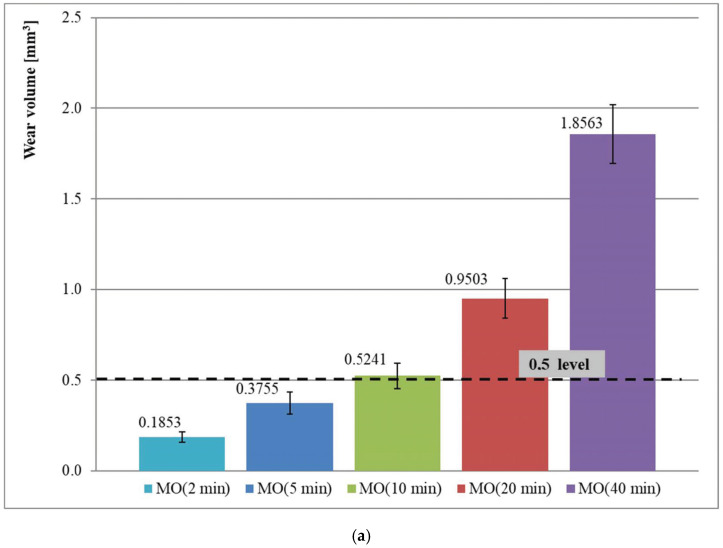

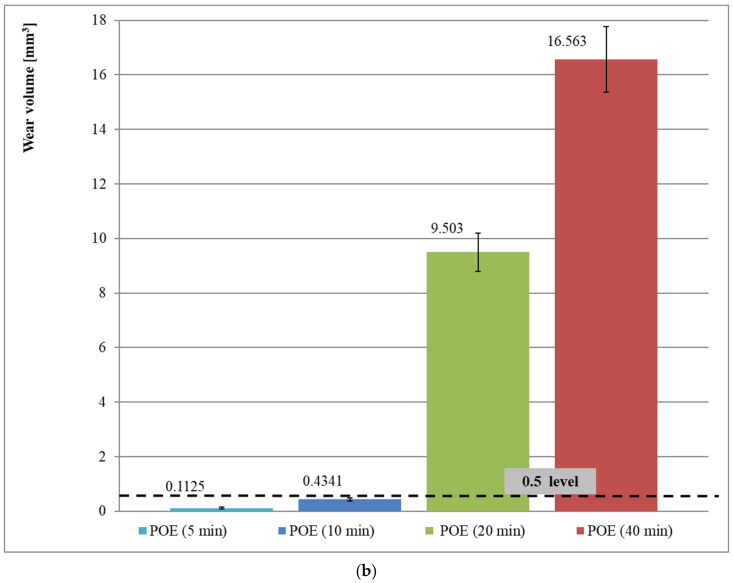
Wear tests duration time selection for mineral (**a**) and polyester (**b**) oils in starved lubrication conditions (p_s_ = 0.21 MPa for mineral oils and 1.10 MPa for polyester oils).

**Figure 3:**
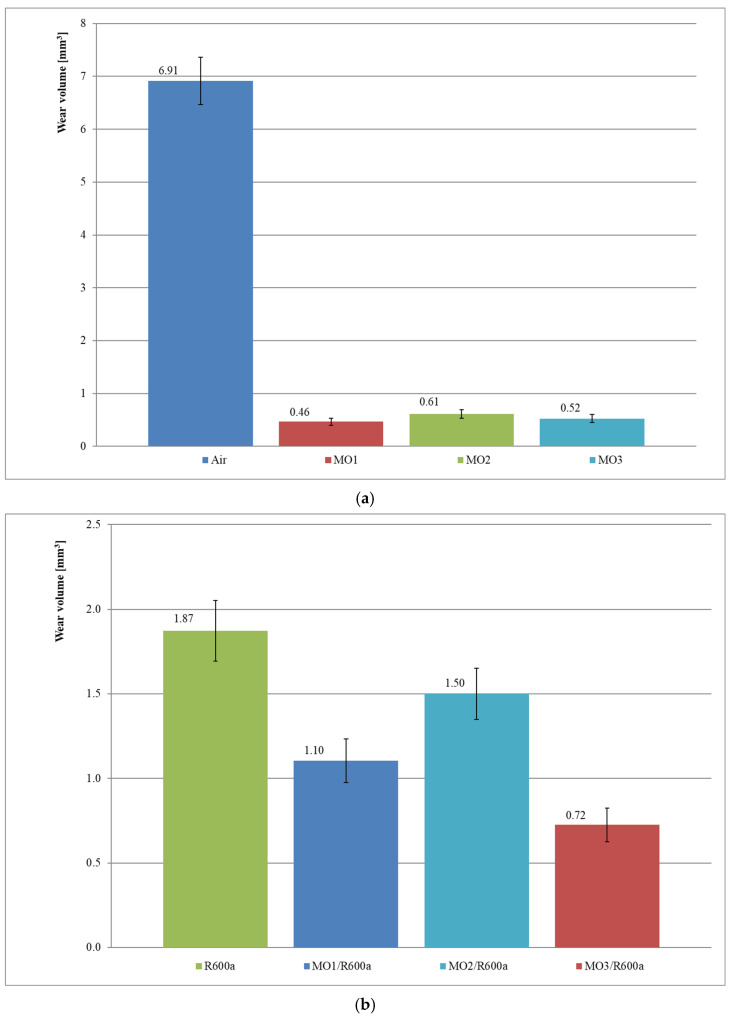
Wear volume results after tests in mineral oils (**a**) and MO/R600a mixtures (**b**) in starved lubrication conditions (series 1–8).

**Figure 4:**
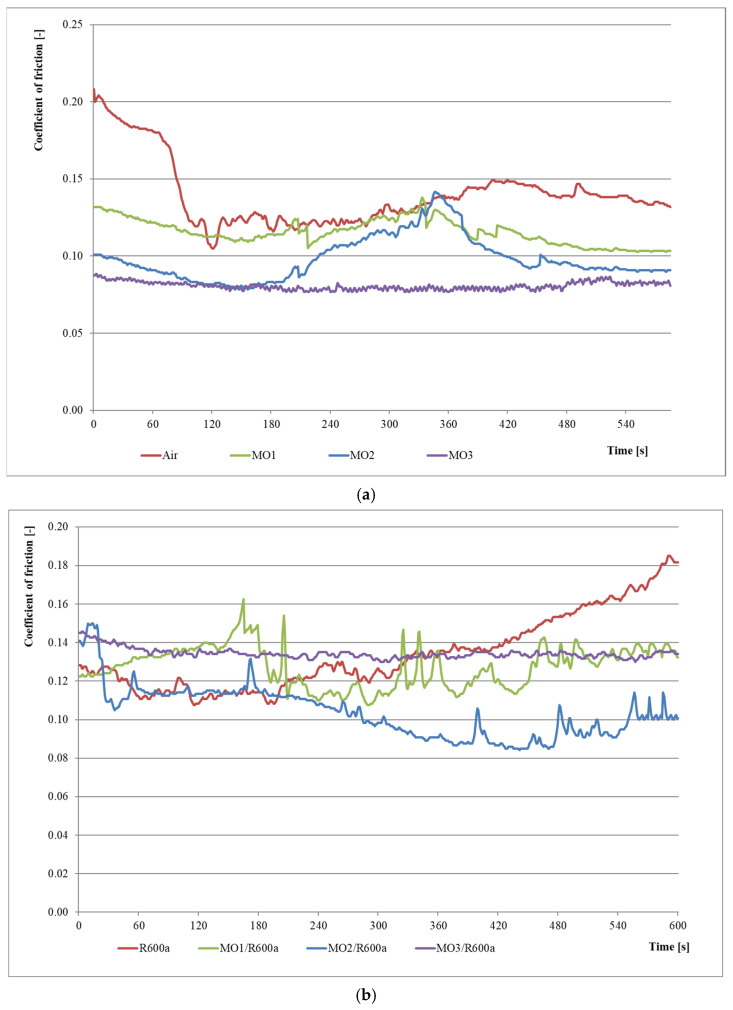
Results for the first eight research series: (**a**) for mineral oils with maximum load (series 1–4); (**b**) for MO/R600a mixtures with maximum load (series 5–8).

**Figure 5:**
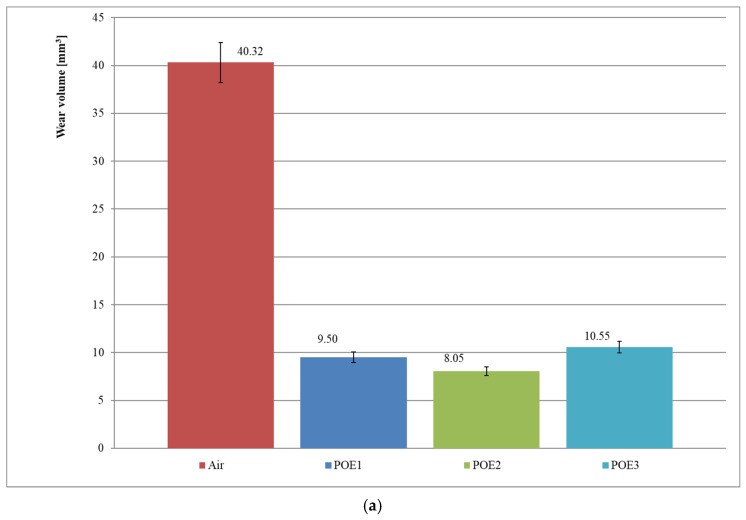

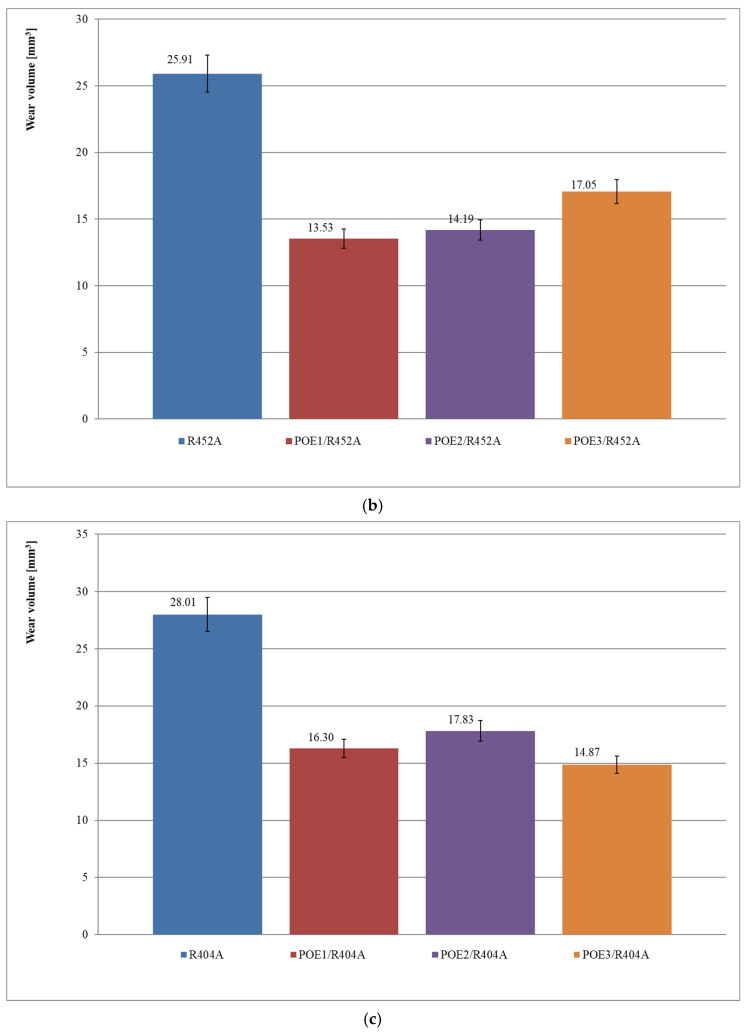
Wear volume results after tests in starved lubrication conditions (series 9–20): (**a**) polyester oils; (**b**) POE/R452A mixtures; (**c**) POE/R404A mixtures.

**Figure 6:**
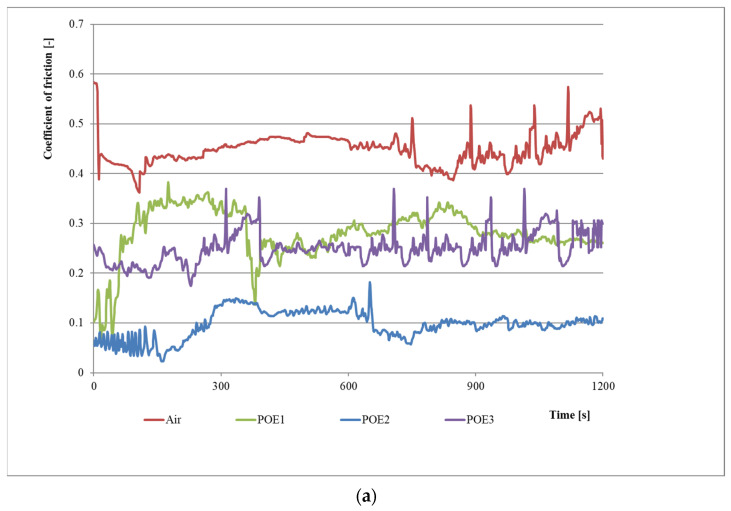

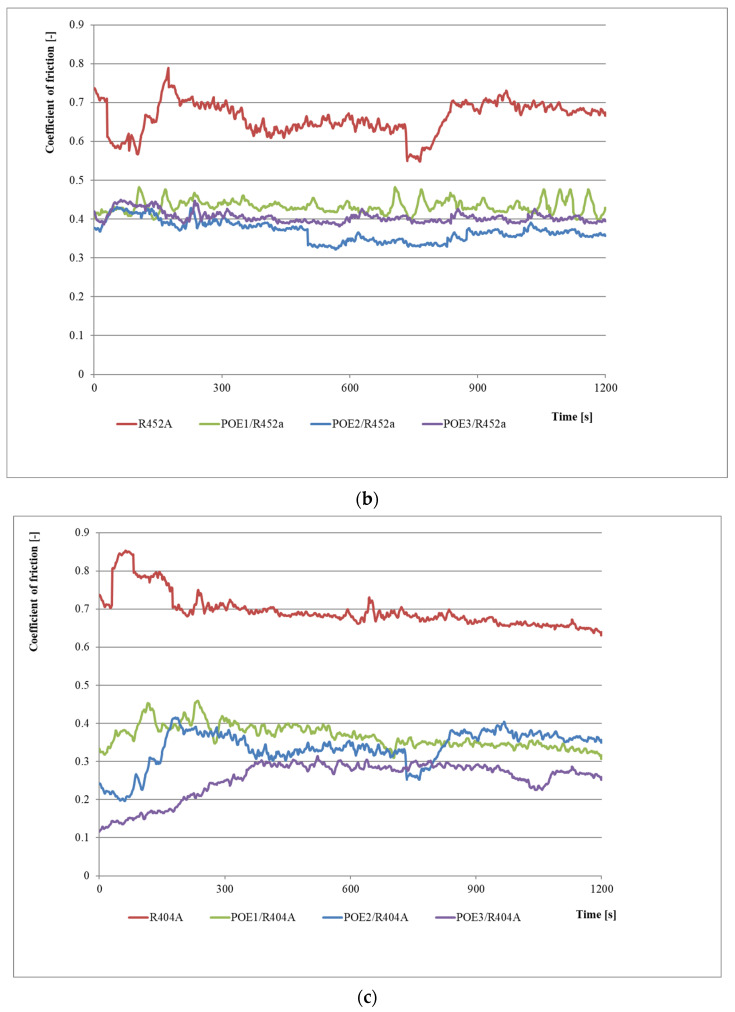
Results for last twelve research series: (**a**) for polyester oils with maximum load (series 9–12); (**b**) for POE/R452a mixtures with maximum load (series 13–16); (**c**) for POE/R404A mixtures with maximum load (series 17–20).

According to the concept proposed by the authors, the wear volume of the block in a block-on-ring friction pair is a measure of the lubricating properties of compressor oils and their mixtures with refrigerant. The evaluation of lubricating properties was preceded by a series of preliminary tests necessary to estimate the duration time of the main wear test (Figure 2). The values of the mean sample wear and their range of variability in the form of standard deviation are presented on the charts illustrating test results (Figure 3 and Figure 5). Other figures shows changes in the coefficient of friction (Figure 4 and Figure 6). 

### 3.1. Wear Test Duration Time 

The wear test duration time (τ_t_) had to be determined in a series of independent tests for each oil group: mineral oils (MO) and polyester oils (POE). Figure 2 presents the results of wear tests for the tested oils under the air pressure in the chamber corresponding to the value of the saturation pressure of the refrigerants that may be used with the lubricants (Table 4) in operational conditions. The duration time of individual tests varied. According to the concept proposed by the authors, the minimum required wear of the sample of 0.5 mm^3^ may be obtained:

- after 10 min for mineral oils dedicated for cooperation with R600a refrigerant (Figure 2a),
- after 20 min for the selected for the tests synthetic polyester oils dedicated for cooperation with R404A and R452A refrigerants (Figure 2b).

### 3.2. Lubricating Properties of MO/R600a Mixture 

The results in Figure 3a present the mean sample wear volume after the initial 4 test series (series 1–4, Table 5). For series 1 (air), the wear volume is 6.91 mm^3^, for series 2 (MO1)—0.46 mm^3^, for series 3 (MO2)—0.61 mm^3^ and for series 4 (MO3)—0.52 mm^3^. The results prove the more than ten times worse lubricating properties of air compared to mineral oils in starved lubrication conditions. Comparing the series 2, 3 and 4, it can be concluded that, in starved lubrication conditions, the tested mineral oils differ in lubricating properties by up to 35% (the comparison concerns the mean sample wear volume). In starved lubrication conditions, MO1 oil has the best lubricating properties, MO3 oil has slightly worse properties and MO2 oil has the worst ones.

The results in Figure 3b present the mean sample wear volume after the tests with the mixtures of mineral oils and R600a refrigerant in starved lubrication conditions (series 5–9, Table 5). For series 5 (R600a), the volume wear is 1.87 mm^3^, for series 6 (MO1/R600a)—1.10 mm^3^, for series 3 (MO2/R600a)—1.50 mm^3^ and for series 4 (MO3/R600a)—0.72 mm^3^. The comparison of the series 5 and 8 proves almost three times better lubricating properties with just one drop of lubricant in a friction pair. Comparing the series 6, 7 and 8, it can be concluded that, in starved lubrication conditions, the tested mineral oils in a mixture with R600a refrigerant differ in lubricating properties even up to 108%. Under the conditions of starved lubrication with an oil–refrigerant mixture, the MO3/R600a mixture has the best lubrication properties, the MO1/R600a mixture has slightly worse properties and the MO2/R600a mixture has the worst ones. 

Based on results of series 1 and 5, the lubricating properties of air are more than three times worse compared to R600a refrigerant in situations where the friction pair lacks lubricant. The relation is contradictory to similar research carried out in [20]. In the paper [20], it was concluded from experimental studies that air provides about two times better lubricating properties than R600a refrigerant alone. Our research indicates that the opposite is true.

Figure 4 shows changes in the coefficient of friction for series 1–8. The waveforms present changes in the coefficient of friction for refrigeration compressor oils in the presence of refrigerant and a small amount of lubricant, which may lead to the formation of an oil–refrigerant mixture under starved lubrication conditions. 

Figure 4a presents changes in the coefficient of friction for series 1–4. It shows that the average value of the coefficient of friction is as follows: for series 1 (air)—0.14, for series 2 (MO1)—0.12, for series 3 (MO2)—0.10 and for series 4 (MO3)—0.08. It can therefore be generally concluded that the coefficient of friction in the presence of just one drop of mineral oils is 14–43% lower than with no lubrication.

Figure 4b illustrates changes of the coefficient of friction for series 5–8. The results show that the mean value of the coefficient of friction is as follows: for series 5 (R600a), series 6 (MO1/R600a) and series 8 (MO3/R600a)—0.13 and for series 7 (MO2/R600a)—0.10. The coefficient of friction in the presence of one drop of mineral oils in a mixture with R600a refrigerant does not change for MO1 and MO3 oils but is approx. 23% lower for MO2 oil. Moreover, for oil–refrigerant mixtures, the coefficient of friction was constant or slightly decreased over time. In contrast, in the absence of oil (series 5), the coefficient of friction increased. 

### 3.3. Lubricating Properties of POE/R404A and POE/R452A Mixtures 

Figure 5a presents the mean sample wear volume after the tests with polyester oils (series 9–12, Table 5). In series 9 (air), the wear volume is 40.32 mm^3^, for series 10 (POE1)—9.50 mm^3^, for series 11 (POE2)—8.05 mm^3^ and for series 12 (POE3)—10.55 mm^3^. The results prove the approximately five times worse lubricating properties of air compared to the tested polyester oils in starved lubrication conditions. Comparing the series 10, 11 and 12, it can be concluded that, in starved lubrication conditions, the tested polyester oils differ in lubricating properties by up to 31%. In starved lubrication conditions, POE2 oil has the best lubricating properties, POE1 oil has slightly worse properties and POE3 oil has the worst ones.

The results in Figure 5b present the mean sample wear volume after the tests with POE/R452A mixtures (series 13–16, Table 5). The wear volume for series 13 (R452A) is 25.91 mm^3^, for series 14 (POE1/R452A)—13.53 mm^3^, for series 15 (POE2/R452A)—14.19 mm^3^ and for series 16 (POE3/R452A)—17.05 mm^3^. A small amount of lubricant in a friction pair provides almost twofold better lubricating properties (series 13 and 14 compared). In starved lubrication conditions, the tested polyester oils in a mixture with R452A refrigerant differ in lubricating properties even up to 26% (series 14–16 compared). Under the conditions of starved lubrication with an oil–refrigerant mixture, the POE1/R452A mixture has the best lubricating properties, the POE2/R452A mixture has slightly worse properties and the POE3/R452A mixture has the worst ones. The results of the series 9 and 13 indicate that the lubricating properties of air are approx. 60% worse than those of R452A refrigerant.

Figure 5c illustrates the mean sample wear volume after the tests with POE/R404A mixtures (series 17–20, Table 5). The wear volume for series 17 (R404A) is 28.01 mm^3^, for series 18 (POE 1/R404A)—16.30 mm^3^, for series 19 (POE2/R404A)—17.83 mm^3^ and for series 20 (POE3/R404A)—14.81 mm^3^. The results prove almost twofold better lubricating properties in the presence of one drop of lubricant in a friction pair (series 17 and 20 compared). Analysing the sample wear for series 9 and 17, it can be concluded that the lubricating properties of air are approx. 44% worse in relation to R404A refrigerant in situations with no lubricant. Comparing the series 18, 19 and 20, it can be concluded that, in starved lubrication conditions, the tested polyester oils in a mixture with R404A refrigerant differ in lubricating properties even up to 20%. Under the conditions of starved lubrication with an oil–refrigerant mixture, the sequence of the tested polyester oils in order of increasing wear (worsening lubricating properties) is as follows: POE1, POE2, POE3. 

The lubricating properties for polyester oils in starved lubrication conditions are 44–60% better compared to no lubrication conditions. Test results for the mixtures of polyester oils and R452A and R404A refrigerants indicate a greater improvement in lubricating properties in the presence of R404A refrigerant (from 41 to 122% compared to 42–76% for R452A refrigerant). It is also worth noting that in no lubrication conditions, the tested refrigerants improved the lubricating properties by approx. 36% for R452A refrigerant and by approx. 30% for R404A refrigerant (compared to dry friction in the presence of air).

Figure 6a presents the coefficient of friction changes for the test series 9–12 with the use of air and polyester oils. The results show that the average value of the coefficient of friction is as follows: for series 9 (air)—0.45, for series 10 (POE1)—0.28, for series 11 (POE2)—0.10 and for series 12 (POE3)—0.25. The coefficient of friction in the starved lubrication of polyester oils may be even more than four times lower than in no lubrication conditions (in the presence of air only—series 9).

Figure 6b presents the coefficient of friction changes for the test series 13–16 with the use of R452A refrigerant and polyester oils. The results show that the average values of the coefficient of friction are as follows: 0.66 for series 13 (R452A), 0.43 for series 14 (POE1/R452A), 0.37 for series 15 (POE2/R452A) and 0.41 for series 16 (POE3/R452A). Generally, the coefficient of friction in starved lubrication condition for MO/R452A mixtures is approx. 50% lower than in no lubrication condition in the presence of R452A refrigerant.

The average coefficient of friction for series 17 (R404A) is 0.70, for series 18 (POE1/R404A)—0.37, for series 19 (POE2/R404A)—0.34 and for series 20 (POE3/R404A)—0.25 (Figure 6c). The results show that the coefficient of friction in a small amount of polyester oils in a mixture with R404A refrigerant is approximately twofold lower than in no lubrication conditions in the presence of R404A refrigerant. 

In evaluating the lubrication efficiency of polyester oils in mixtures with R452A and R404A refrigerants, it should be noted that the tested oils improve the cooperation conditions more in the presence of R404A refrigerant (even about a twofold decrease in value). In the case of R452A refrigerant, polyester oils improved cooperation conditions by approx. 50%.

## 4. Discussion

The test results indicate that even a small amount of oil in a friction pair significantly changes the nature of interactions (lowers the coefficient of friction and reduces wear). Such regularity was observed for all the tested oil–refrigerant mixtures (Figure 3 and Figure 5).

The test results presented in the article show that, in starved lubrication conditions, refrigerant changes the lubricating properties of compressor oil by forming a mixture with the oil. The test procedure proposed by the authors provides the basis for identifying the changes in quantitative terms (for example comparing Figure 3a,b). 

Changes in lubricating properties of oil–refrigerant mixtures compared to the oils alone have been found for all the analyzed mixtures (Figure 3 and Figure 5). In the case of the tested mineral oils, the presence of R600a refrigerant caused deterioration in lubricating properties as outlined below (Figure 3):

- 140% for MO1 oil (series 2 and 6 compared),
- 146% for MO2 oil (series 3 and 7 compared),
- 38% for MO3 oil (series 4 and 8 compared).

The increase of the mean wear volume (deterioration of lubricating properties) for polyester oils is as follows (Figure 5):

- In a mixture with R452A:
  - 42% for POE1 oil (series 10 and 14 compared),
  - 76% for POE2 oil (series 11 and 15 compared),
  - 62% for POE3 oil (series 12 and 16 compared),
- In a mixture with R404A
  - 71% for POE1 oil (series 10 and 18 compared),
  - 122% for POE2 oil (series 11 and 19 compared),
  - 41% for POE3 oil (series 12 and 20 compared).

The worsened lubricating properties of compressor oil in the presence of refrigerant are compatible with general expectations [4,18,19,21,24]. Such a trend under starved (or submerged) lubrication conditions was found for the R404A/POE [3,26,49] and for R600a/MO mixtures [13,20,28]. The reason for the deterioration of lubricating properties is the formation of the refrigerant–oil mixture which has a lower viscosity than oil. In the case of zeotropic refrigerants (R404A, R452A), a mixture is formed by natural convection. This process is determined by a higher density of the refrigerant than the oil (Table 1 and Table 2). In this case, the formation of a saturated mixture is quite fast (short mixing time—Table 4). For the natural refrigerant R600a, a mixture is formed by purely intermolecular diffusion interactions. For MO/R600a mixtures, the process is relatively long. For both mechanisms of oil–refrigerant mixture formation under starved lubrication conditions, a clear deterioration (at least tens of percent) of lubricating properties was found in comparison with oil. Daniel plots can be used to describe the properties of the oil—refrigerant mixture under refrigeration compressor operating conditions. They indicate the solubility of the refrigerant in the respective lubricant and the viscosity of the mixture in accordance with temperature and pressure. Daniel plots are not always available, especially for new refrigerants. The advantage of the proposed method for evaluating lubricating properties is the fact that is makes it possible to determine the direct effect of change in lubricant viscosity in the form of wear volume. The measured wear volume is the “response” to a change in oil viscosity for a specific material pair in a specific friction node. The model block-on-ring friction node used in the study (Figure 1a) corresponds in geometry to the actual connecting rod—crankshaft association in refrigeration compressors. 

Additionally, the proposed method makes it possible to compare the lubricating properties of oil–refrigerant mixtures in starved lubrication conditions for groups of oils with the same viscosity grade that are substitutes (operational alternatives). Table 6 presents the rankings of lubricating properties for all the tested lubricants. 

Oils and oil–refrigerant mixtures have been classified according to the increasing sample wear (decreasing lubricating properties). For both tested types of oils, it was found that the ranking of lubricating properties for oils differs from the ranking of lubricating properties for oil–refrigerant mixtures. For all the analyzed cases (Table 6), the leader of the ranking was also different. The obtained results show differences in the lubricating properties of oil–refrigerant mixtures for oils that are alternatives intended for interchangeable use in systems with a given refrigerant. The results also prove that the lubricating properties of oil–refrigerant mixtures in starved lubrication conditions cannot be effectively assessed based on the lubricating properties of the oil. The influence of refrigerant on the lubricating properties of oil in a mixture is clear and is a unique feature of the oil–refrigerant mixture. It is worth noting that the article presents test results for various types of oil–refrigerant mixtures: for the natural refrigerant R600a with mineral oils and the synthetic refrigerants R404A and R452A with synthetic polyester oils.

In order to look for the reasons for the change in lubricating properties, an assessment of the oils’ chemical composition was carried out. The results (Raman spectra) are presented in the work [30]. Performed analysis did not indicate significant differences in chemical composition in the tested groups of oils (POE and MO). All the oils in each group contain the same hydrocarbon base. Only in POE1 oil was the presence of an additive found—sulfonate dispersant. Presumably, under submerged lubrication conditions this additive guaranteed the best lubricating properties for R404A/POE1 and R452A/POE1 mixtures (results presented in an earlier publication [30]). The mentioned additive prevents wear products from combining into large-sized assemblies. Uniform distribution of wear products in the oil volume could significantly reduce abrasive wear. Agglomerates of wear products intensify abrasive interactions. The rankings shown in Table 6 indicate that under starved lubrication conditions, the found additive no longer provides POE1 oil with an advantage over POE2 and POE3 oils. Therefore, it is necessary to continue research on the identification of oil components and additives to improve the lubricating properties of the oil–refrigerant mixture formation under starved lubrication conditions.

The method of evaluating lubricating properties can be effective in the comparative analysis of a group of oils that are substitutes (alternative lubricants). The obtained rankings of oils by wear volume (Table 6) can provide a good basis for the selection of a lubricant for refrigeration compressors (especially for new, environmentally friendly refrigerants, e.g., R452A). It is worth noting that different rankings of lubricating properties for oil–refrigerant mixtures for each of the refrigerants, R404A and R452A, have been found in the case of polyester oils. The experience of using the given oil with some refrigerant cannot constitute a sound basis for the presumption that it is suitable for use in a refrigeration system with a new refrigerant. The polyester oils tested in starved lubrication conditions showed better lubricating properties with the previously used R404A refrigerant than with the more ecological R452A refrigerant. This indicates the need to search for more effective formulations of polyester oils for applications with R452A refrigerant. In addition, the use of oils dedicated to R404A refrigerant in systems filled with R452A may cause worse lubrication and reduced durability of refrigeration compressor friction pairs due to deteriorated lubricating properties in starved lubrication conditions.

**Figure 7:**
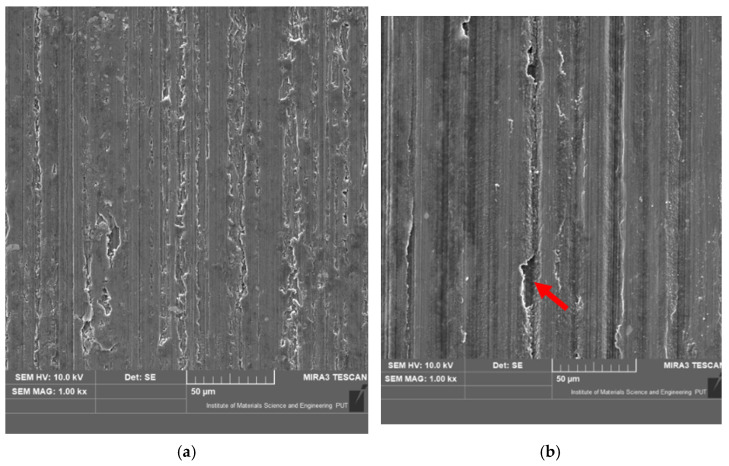

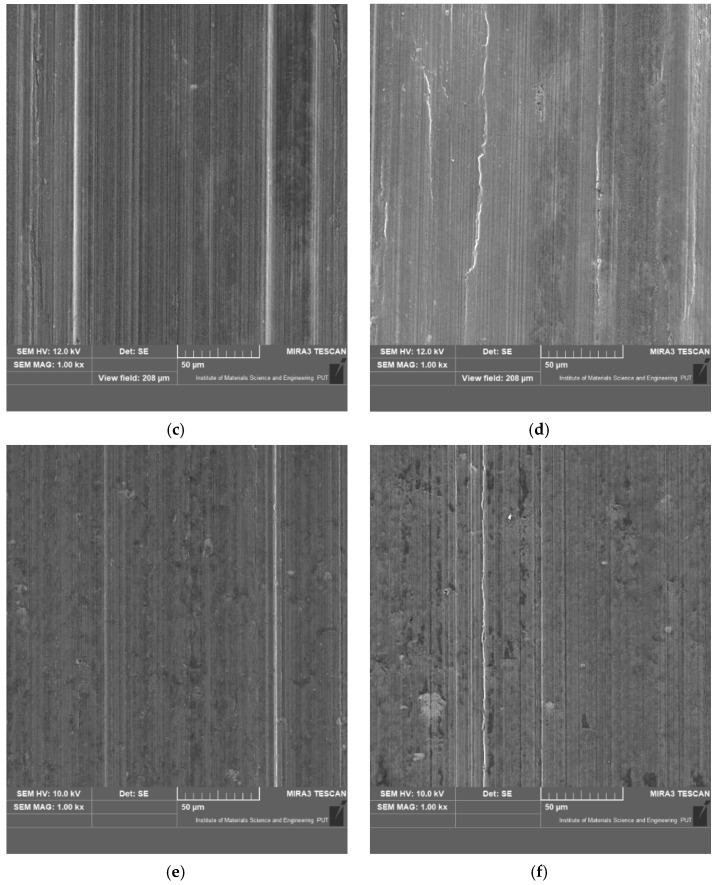
Exemplary microscopic observation of wear traces on the sample surface: (**a**) full lubrication in POE3/R452A; (**b**) starved lubrication in POE3/R452A; (**c**) full lubrication in POE3/R404A; (**d**) starved lubrication in POE3/R404A; (**e**) full lubrication in MO/R600a; (**f**) starved lubrication in MO/R600a.

After finishing the tests, microscopic observation of wear traces on the sample surface was made. Images were taken with a Tescan Mira3 electron microscope. The purpose of the analysis was to identify the wear mechanisms. Exemplary images are presented in Figure 7. The images depict after-test wear traces for all the tested oil–refrigerant mixtures. They are arranged in pairs to illustrate the effects of wear in the conditions of full (sufficient amount of oil which filled half of the test chamber) and starved lubrication (small amount of oil—a drop on a friction surface). Another series of images documents tests in full lubrication conditions described in [49].

Abrasive wear is the dominant form of wear in all the cases presented in Figure **7**. It is indicated by parallel grooves on the surface of the wear trace. For starved lubrication, the grooves are more numerous and deeper. This is confirmed by the fact that with a small amount of oil in a friction pair, more frequent contact of the roughness protrusions of both interacting surfaces takes place and material removal is more intensive as a result of mechanical interactions.

Figure 7 illustrates, inter alia, the wear trace after tests with POE3 oil and two different refrigerants—R452A (Figure 7a,b) and R404A (Figure 7c,d). The images clearly indicate that, under starved lubrication conditions, the wear mechanisms are more intense for the POE3/R452A mixture. This corresponds to the wear volume results presented in Figure 5. After the tests in the POE3/R452A mixture (Figure 7b), the grooves are deeper and there is more material detachment (indicated by red arrow) than after the tests in the POE3/R404A mixture (Figure 7d). 

The microscopic analysis of the sample wear trace (Figure 7) additionally emphasizes the need for individual selection of a compressor oil compatible with a given refrigerant by carrying out tests in conditions reflecting the actual operational conditions (lubricating friction pairs with an oil–refrigerant mixture). The method proposed by the authors makes such an assessment possible.

## 5. Conclusions

1. The results show that even a small amount of compressor oil in the node’s contact area clearly changes the nature of the interactions; it lowers the coefficient of friction and reduces wear. Unfortunately, the presence of refrigerant significantly deteriorates the lubricating properties of oil (decrease of 40–120%). This change is caused by the formation of an oil–refrigerant mixture. The obtained mixture has a lower viscosity than oil.
2. Among oils, that are an alternative for use with particular refrigerants, the significant differentiation of lubricating properties of oil–refrigerant mixtures was shown. For the studied oils, it was found that the ranking of the lubricating properties of oils differs from the ranking of the lubricating properties of oil—refrigerant mixtures. In all the analyzed cases, the leader of this ranking also changes (Table 6).
3. In order to evaluate and properly select compressor oils for refrigerant, the lubricating properties should be tested in a mixture with the refrigerant under conditions similar to actual operation. Such an evaluation of lubricating properties is made possible by the author’s method of testing the wear of the block-on-ring friction node. The obtained rankings of lubricating properties for oils (due to the wear volume) can provide good guidelines for the proper selection of a lubricant for refrigeration compressors (especially for new, environmentally friendly refrigerants, such as R452A).

## Figures and Tables

**Table 1:** Properties of investigated polyester oils.

Oil Type	Property				
	Kinematic Viscosity [mm^2^ s^−1^]		Density at 15 °C [kg m^−3^]	Flash Point [°C]	Pour Point [°C]
	40 °C	100 °C			
MO1	32	4.8	889	185	−45
MO2	29.5	4.31	909	178	−40
MO3	32	4.4	910	190	−42
POE1	32	6.0	981	250	−54
POE2	31.2	6.0	1006	243	−48
POE3	32	6.1	1007	240	−48

**Table 2:** Properties of investigated refrigerants.

Property	Refrigerant		
	R600a	R404A	R452A
Composition	C4H10	R125/R143/R134A	R125/R32/R1234yf
Mass percentage	100	44/52/4	59/11/30
Boiling point (°C) at 1 kPa	−0.5	−46.6	−47.2
Critical pressure (kPa)	3796	3728	4014
Critical temperature (°C)	151.98	72.1	75.05
Density at 25 °C [kg m^−3^]	551	1048	1125,5
ODP	0	0	0
GWP	3	3943	1945
Class	A3	A1	A1
Lubricant type	MO	POE	POE
Evaporator pressure (kPa)	80.20	183.30	701.90
Condenser pressure (kPa)	535.40	2197.50	2903.70

**Table 3:** Parameters selection procedure.

Selection Order	Parameter			Substance			Selection Procedure	
	Pressure in Chamber (p_s_)	Wear Tests Duration Time (τ_t_)	Mixture Production Time (τ_m_)	Refrigerant	Oil	Oil–Refrigerant Mixture	Data	Criterion
1	Selection			X			Lg p-h chart	Saturation pressure in room temperature
2	Constant—selected in step 1 (air)	Selection			X		Results of wear volume after different tests duration time (τ_t_)	Wear bigger than 0.5 mm^3^
3	Constant—selected in step 1 (refrigerant)	Constant—selected in step 2	Selection			X	Results of wear volume after different mixture production time (τ_m_)	Wear volume stabilizes

**Table 4:** Individually selected parameters for oil/refrigerant mixtures in starved lubrication conditions.

Parameter	Unit	MO/R600a	POE/R404A	POE/R452A
Sliding velocity	[m/s]	0.5	0.5	0.5
Friction node load	[N]	120	120	120
Amount of lubricant	mg	30 (1 drop)	30 (1 drop)	30 (1 drop)
Way of oil—refrigerant mixture preparation	-	Without limiting supply of refrigerant	Without limiting supply of refrigerant	Without limiting supply of refrigerant
Refrigerant pressure p_s_	MPa	0.21	1.10	1.20
Wear tests duration time τ_t_	[min]	10	20	20
Oil—refrigerant mixture formation time τ_m_	[min]	1200	40	40

**Table 5:** Summary of research series.

Series Number	Lubricant
1	Air (τ_t_ = 10 min)
2	MO1
3	MO2
4	MO3
5	R600a
6	MO1/R600a
7	MO2/R600a
8	MO3/R600a
9	Air (τ_t_ = 20 min)
10	POE1
11	POE2
12	POE3
13	R452A
14	POE1/R452A
15	POE2/R452A
16	POE3/R452A
17	R404A
18	POE1/R404A
19	POE2/R404A
20	POE3/R404A

**Table 6:** Rankings of lubricating properties for all the tested lubricants.

Lubricant	Rankings of Lubricating Properties		
	1	2	3
MO	MO1	MO3	MO2
MO/R600a	MO3	MO1	MO2
POE	POE2	POE1	POE3
POE/R404A	POE3	POE1	POE2
POE/R452A	POE1	POE2	POE3

## Data Availability

Not applicable.

## References

[1] Abas N., Kalair A.R., Khan N., Haider A., Saleem Z., Saleem M.S. (2018). Natural and synthetic refrigerants, global warming: A review. Renew. Sustain. Energy Rev..

[2] Calm J.M. (2008). The next generation of refrigerants—Historical review, considerations, and outlook. Int. J. Refrig..

[3] Bogdanovská G., Molnár V., Fedorko G. (2018). Failure analysis of condensing units for refrigerators with refrigerant R134a, R404A. Int. J. Refrig..

[4] Bhutta M., Khan Z., Garland N., Ghafoor A. (2018). A Historical Review on the Tribological Performance of Refrigerants used in Compressors. Tribol. Ind..

[5] Stachowiak G., Batchelor A.W. (2013). Engineering Tribology.

[6] Bhutta M.U., Khan Z.A., Garland N. (2018). Wear Performance Analysis of Ni–Al2O3 Nanocomposite Coatings under Nonconventional Lubrication. Materials.

[7] Davim J.P. (2011). Tribology for Engineers A Practical Guide.

[8] Davim J.P. (2017). Progress in Green Tribology: Green and Conventional Techniques.

[9] Zhang Z., Shao F., Liang Y., Lin P., Tong X., Ren L. (2017). Wear Behavior of Medium Carbon Steel with Biomimetic Surface Under Starved Lubricated Conditions. J. Mater. Eng. Perform..

[10] Macián V., Tormos B., Bermúdez V., Bastidas S. (2021). Development of a floating liner test rig and lubrication model for the study of the piston compression ring friction force under fully flooded and starved lubrication. Tribol. Int..

[11] Akram M.W., Polychronopoulou K., Polycarpou A.A. (2013). Lubricity of environmentally friendly HFO-1234yf refrigerant. Tribol. Int..

[12] Akram M.W., Polychronopoulou K., Polycarpou A.A. (2014). Tribological performance comparing different refrigerant–lubricant systems: The case of environmentally friendly HFO-1234yf refrigerant. Tribol. Int..

[13] Birol Y., Birol F. (2008). Sliding wear behaviour of thixoformed AlSiCuFe alloys. Wear.

[14] De Mello J., Binder R., Demas N., Polycarpou A. (2009). Effect of the actual environment present in hermetic compressors on the tribological behaviour of a Si-rich multifunctional DLC coating. Wear.

[15] Garland N.P., Hadfield M. (2005). Retracted: Tribological analysis of hydrocarbon refrigerants applied to the hermetic compressor. Tribol. Int..

[16] Garland N., Hadfield M. (2005). Environmental implications of hydrocarbon refrigerants applied to the hermetic compressor. Mater. Des..

[17] Hong-Gyu J., Se-Doo O., Young-Ze L. (2009). Friction and wear of the lubricated vane and roller materials in a carbon dioxide re-frigerant. Wear.

[18] Mizuhara K., Akei M., Matsuzaki T. (1994). The friction and wear behaviour in controlled alternative refrigerant atmosphere. Tribol. Trans..

[19] Na B.C., Chun K.J., Han D.-C. (1997). A tribological study of refrigeration oils under HFC-134a environment. Tribol. Int..

[20] Sariibrahimoglu K., Kizil H., Aksit M.F., Efeoglu I., Kerpicci H. (2010). Effect of R600a on tribological behavior of sintered steel under starved lubrication. Tribol. Int..

[21] Sheiretov T., Van Glabbeek W., Cusano C. (1995). Simulative friction and wear study of retrofitted swash plate and rolling piston compressors. Int. J. Refrig..

[22] Suha A.Y., Patel J.J., Polycarpou A.A., Conry T.F. (2006). Scuffing of cast iron and Al390-T6 materials used in compressor applica-tions. Wear.

[23] Tanaka M., Matsuura H., Taira S., Nakai A. Selection of a refrigeration oil for R32 refrigerant and evaluation of the com-pressor reliability. Proceedings of the International Compressor Engineering Conference 2014.

[24] Yoon H., Sheiretov T., Cusano C. Tribological evaluation of various aluminum alloys in lubricant/refrigerant mixtures. Proceedings of the International Compressor Engineering Conference 1996.

[25] Chen Z., Wu J., Li G. (2018). Experimental study on the tribological characteristic of vane–roller interface of HC290 rotary compressor with mineral oil. Int. J. Refrig..

[26] Matsumoto T., Kaneko M., Kawaguchi Y. (2017). Evaluations of PVE Lubricants for Refrigeration and Air Conditioning system with the Low GWP Refrigerants. IOP Conf. Ser. Mater. Sci. Eng..

[27] Bhutta M.U., Khan Z.A. (2020). Wear and friction performance evaluation of nickel based nanocomposite coatings under refrigerant lubrication. Tribol. Int..

[28] Górny K., Stachowiak A., Tyczewski P., Zwierzycki W. (2016). Evaluation of lubricating properties of mixtures of mineral oils with refrigerant R600a. Tribologia.

[29] Górny K., Tyczewski P., Zwierzycki W. (2014). Description of the experimental method and procedure of model wear test of re-frigeration compressors’ parts. Solid State Phenom..

[30] Górny K., Stachowiak A., Tyczewski P., Zwierzycki W. (2016). Lubricity evaluation of oil–refrigerant mixtures with R134a and R290. Int. J. Refrig..

[31] Górny K., Stachowiak A., Tyczewski P., Zwierzycki W. (2016). Research Idea and Methodology for Determining Test Parameters for the Lubricity Evaluation of Oil/Refrigerant Mixtures. Tribologia.

[32] Górny K., Stachowiak A., Tyczewski P., Zwierzycki W. (2017). Effect of flushing fluid addition on lubricity of refrigerant compressor oils. Tribologia.

[33] Mishra S.P., Polycarpou A.A. (2011). Tribological studies of unpolished laser surface textures under starved lubrication conditions for use in air-conditioning and refrigeration compressors. Tribol. Int..

[34] Bai L., Meng Y., Khan Z.A., Zhang V. (2017). The Synergetic Effects of Surface Texturing and MoDDP Additive Applied to Ball-on-Disk Friction Subject to Both Flooded and Starved Lubrication Conditions. Tribol. Lett..

[35] Akram M.W., Polychronopoulou K., Seeton C., Polycarpou A.A. (2013). Tribological performance of environmentally friendly refrigerant HFO-1234 yf under starved lubricated conditions. Wear.

[36] Zeng F., Liu Y., Shao F., Li X., Yu Z., Guo Y., Wan Z., Lu L., Zhang Z. (2020). Wear Behavior of Medium-Carbon Steel with Different Laser-Textured Densities under Starved Lubrication. Coatings.

[37] Saeidi F., Parlinska-Wojtan M., Hoffmann P., Wasmer K. (2017). Effects of laser surface texturing on the wear and failure mechanism of grey cast iron reciprocating against steel under starved lubrication conditions. Wear.

[38] Akram M.W., Polycarpou A.A. (2015). Wear Mechanisms of Gray Cast Iron in the Presence of Environmentally Friendly Hydro-fluoroolefin-Based Refrigerant and the Effect of Tribofilm Formation. J. Tribol..

[39] Hu X., Zhang Z., Yao Y., Wang Q. (2018). Non-azeotropic refrigerant charge optimization for cold storage unit based on year-round performance evaluation. Appl. Therm. Eng..

[40] Bortolini M., Gamberi M., Gamberini R., Graziani A., Lolli F., Regattieri A. (2015). Retrofitting of R404a commercial refrigeration systems using R410a and R407f refrigerants. Int. J. Refrig..

[41] Heredia-Aricapa Y., Belman-Flores J., Mota-Babiloni A., Serrano-Arellano J., García-Pabón J.J. (2019). Overview of low GWP mixtures for the replacement of HFC refrigerants: R134a, R404A and R410A. Int. J. Refrig..

[42] Mendoza-Miranda J.M., Mota-Babiloni A., Navarro-Esbrí J. (2016). Evaluation of R448A and R450A as low-GWP alternatives for R404A and R134a using a micro-fin tube evaporator model. Appl. Therm. Eng..

[43] Mota-Babiloni A., Makhnatch P., Khodabandeh R. (2017). Recent investigations in HFCs substitution with lower GWP synthetic alternatives: Focus on energetic performance and environmental impact. Int. J. Refrig..

[44] Makhnatch P., Mota-Babiloni A., Rogstam J., Khodabandeh R. (2017). Retrofit of lower GWP alternative R449A into an existing R404A indirect supermarket refrigeration system. Int. J. Refrig..

[45] Devecioglu ·A.G., Oruç V. (2021). Experimental comparison of R404A and R452A in refrigeration systems. Sci. Technol. Built Environ..

[46] Li G. (2017). Comprehensive investigation of transport refrigeration life cycle climate performance. Sustain. Energy Technol. Assess..

[47] Saleem A., Kim M.-H. (2021). Miscibility analysis of polyol-ester based oil SW32 with R404A and low-GWP refrigerant R452A. Int. J. Refrig..

[48] Altinkaynak M. (2021). Exergetic performance analysis of low GWP alternative refrigerants for R404A in a refrigeration system. Int. J. Low-Carbon Technol..

[49] Górny K., Stachowiak A., Tyczewski P., Zwierzycki W. (2018). Lubricity of selected oils in mixtures with the refrigerants R452A, R404A, and R600a. Tribol. Int..

[50] Halon T., Gil B., Zajaczkowski B. (2022). Comparative investigation of low-GWP binary and ternary blends as potential replacements of HFC refrigerants for air conditioning systems. Appl. Therm. Eng..

[51] Sawczuk W., Ulbrich D., Kowalczyk J., Merkisz-Guranowska A. (2021). Evaluation of Wear of Disc Brake Friction Linings and the Variability of the Friction Coefficient on the Basis of Vibroacoustic Signals. Sensors.

